# The age of the target cell affects B-cell leukaemia malignancy

**DOI:** 10.18632/aging.100244

**Published:** 2010-12-11

**Authors:** Carolina Vicente-Dueñas, Fernando Abollo-Jiménez, Lucía Ruiz-Roca, Esther Alonso-Escudero, Rafael Jiménez, María Begoña García Cenador, Francisco Javier García Criado, César Cobaleda, Isidro Sánchez-García

**Affiliations:** ^1^ Experimental Therapeutics and Translational Oncology Program, Instituto de Biología Molecular y Celular del Cáncer, CSIC/ Universidad de Salamanca, Campus M. de Unamuno s/n, 37007- Salamanca, Spain; ^2^ Departamento de Fisiología y Farmacología, Universidad de Salamanca, Edificio Departamental, Campus M. de Unamuno s/n, 37007- Salamanca, Spain; ^3^ Departamento de Cirugía, Universidad de Salamanca, Spain; ^4^ Centro de Biología Molecular Severo Ochoa, CSIC/Universidad Autónoma, Madrid, Spain

**Keywords:** B-cell leukaemia, age, target cell, BCR-ABL

## Abstract

The incidence, malignancy and treatment resistance of many types of human B-cell leukaemias (B-ALL) are directly related to patient age. A major obstacle to elucidate the contribution of age to the development and evolution of leukaemias is the lack of appropriate mouse models where precise control of the timing of oncogene expression is possible. Here we present proof-of-principle experiments showing how a conditional transgenic mouse model of BCR-ABLp190-driven B-ALL offers the opportunity to test the hypothesis that the age of the leukemic cells-of-origin of B-ALL influences B-ALL malignancy. B-ALLs generated from 12- and 20-month-old progenitors gave rise to a more invasive B-ALL than the one developed from 4-month old precursors. This was evidenced by survival analysis revealing the increased malignancy of B-ALLs generated from 20 or 12-month-old transformed progenitors compared with the 4-month equivalents (median survival of 88 days versus 50.5 and 33 days, respectively). Our study shows that the age of target cells at the time of transformation affects B-ALL malignancy.

## INTRODUCTION

An improved understanding of B cell lineage acute lymphoblastic leukaemia (B-ALL) pathophysiology has been gained in the process of identification of the genetic abnormalities consistently present in B-cell blasts [1-3]. A rather limited variety of gene fusions created by chromosomal translocations are involved in the genesis of B-ALL: basically *TEL-AML1*, *MLL* rearrangements, *BCR-ABL* and *E2A-PBX1* [2,4]. However, the frequency of genetically defined leukaemia subtypes differs between children and adults: for example, TEL-AML^+^ leukaemias are almost exclusively present in children (22% of ALLs vs 2% in the adults) while BCR-ABL^+^ ALLs are much more frequent in adults (25% vs. 3% in children) [2,4,5]. BCR-ABL^+^(and, in most of the cases, specifically the BCR-ABLp190 protein form) represents the most frequent cytogenetic abnormality (25-30% of cases) in adults and defines the B-ALL subset with the most unfavorable prognosis [6]. Increased age has a negative impact on human B-ALL survival and directly correlates with increased incidence, malignancy, and treatment resistance [3]. However, little is known about age-related mechanisms that impact B-ALL malignancy. There are many evidences indicating that aging has a clear impact both in the numbers and in the functionality of stem cells [7-12] and B lymphoid progenitors [13-15]. It has been described that BCR-ABLp190^+^ leukaemias have to originate from early progenitors/stem cells, given the fact that the oncogene cannot by itself endow the target cell with stem cell properties [16]. The same conclusion is supported by transplantation experiments of human B-ALL purified subpopulations into immunocompromised mice [4,17,18]. Therefore, any impact that age might have on the target cell-of-origin population may reflect in the characteristics of the leukemic disease.

A major obstacle to elucidate the contribution of age to the development and evolution of leukaemias is the lack of appropriate mouse models where precise control of the timing of oncogene expression is possible. Here we present proof-of-principle experiments showing how a conditional transgenic mouse model of BCR-ABLp190-driven B-ALL offers the opportunity to evaluate the impact of age-related mechanisms in B-ALL malignancy.

**Figure 1.**
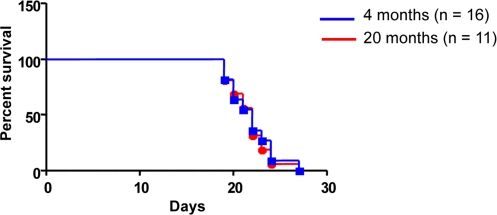
Host age does not affect survival outcome To determine the impact of host age on survival, 1 x 10^5^ cells of a characterized B-ALL cell line Ba/F3-p190 (REF) were IV injected into syngeneic host mice aged 4 months (n = 16) or 20 months (n = 11). Kaplan-Meier survival plot demonstrates no difference in survival (log rank test *P* = 0.9011).

**Figure 2.**
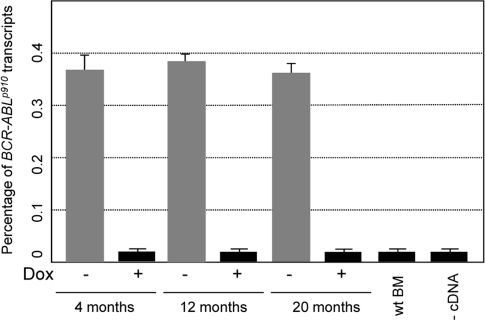
Transformed hematopoietic pro-genitor/stem cells express comparable levels of exogenous *BCR-ABL* Expression of *BCR-ABL* was measure by real-time PCR in BM cells of CombitTA-p190 mice at the age of 4 months, 12 months, and 20 months. Doxycycline was given at 4mg/mL for 4 months, 12 months, and 20 months. *BCR-ABL expression* was measured in CombitTA-p190 mice under doxycycline treatment (+) and mice of the same age once the doxycycline has been removed (−). The mean Ct values of triplicate assays are presented. (BM, bone marrow; wt, wild-type).

## RESULTS AND DISCUSSION

### Survival of BCR-ABL^+^ ALL cells is cell-autonomous and independent of the host's age

To determine the impact of host age on the survival of BCR-ABL^+^ leukemic cells, 1 x 10^5^ cells of a characterized B-ALL cell line Ba/F3-p190 [19] were injected intravenously into syngenic host mice of 4 (n = 16) or 20 (n = 11) months of age. Injected mice started to die due to leukemic infiltration (data not shown) around 20 days post-injection. Kaplan-Meier survival plot demonstrates that there is no difference in survival between young or old recipient mice (log rank test *P* = 0.9011) (Figure 1). These results indicate that host age does not influence BCR-ABL^+^ ALLs malignancy.

### Age-dependent malignancy of transformed hematopoietic progenitor cells *in vivo*

As mentioned, BCR-ABLp190^+^ ALLs have been identified as being originated in cells with the characteristics of a stem cell, and there are many evidences indicating that aging affects both the numbers and the functionality of stem cells and B lymphoid progenitors. Therefore, to investigate if the age of the target cells also has an impact in malignant development we have taken advantage of the doxycline-controlled transgenic CombitTA-BCR-ABLp190 mouse line [20] to induce the expression of the leukaemia-triggering oncogene in mice of different ages. The CombitTA-BCR-ABL-p190 mice expressing the *BCR-ABL^p190^*chimeric gene product have previously been described to consistently show the B-ALL pathologic phenotype with which this oncogene is associated in humans [20]. In the present work, BCR-ABLp190 expression was activated in CombitTA-BCR-ABLp190 mice of different ages (4-, 12- and 20-month, respectively) by removing the doxycycline from the drinking water. All mice demonstrated equivalent levels of exogenous BCR-ABL expression, thus precluding any effect from transgene expression changes with age (Figure 2).

**Figure 3.**
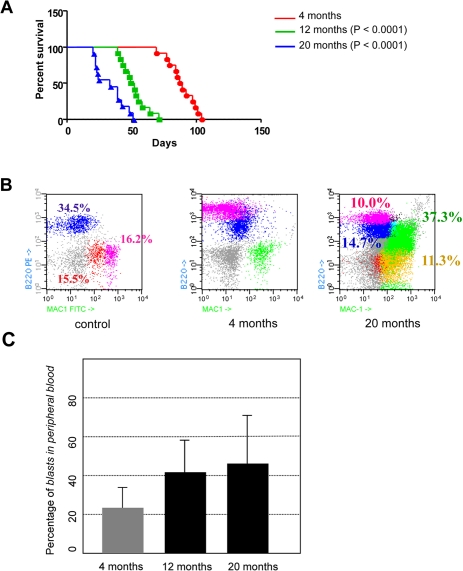
Age-dependent malignancy of transformed hematopoietic progenitor cells in vivo. (**A**) Kaplan-Meier survival analysis. Animals where BCR-ABL expression was induced at 4-month of age survived significantly longer than animals where BCR-ABL expression was induced at 12- or 20-month of age (median survival of 88 days versus 50.5 and 33 days, respectively; log rank *P* < 0.0001 and 0.0001, respectively). (**B**) CombitTA-p190 mice were evaluated for disease progression by flow citometry. Cells from peripheral blood of CombitTA-p190 and control mice were analyzed by flow cytometry with combination of the specific myeloid (Mac1) and B-cell lymphoid (B220) markers. A representative flow cytometry analysis is shown Characteristic blast cells in BCR-ABL B-cell leukaemia co-expressed B-cell and myeloid markers indicated by the presence of Mac^+^B220^+^ cells. (**C**) Variability on the percentage of blast cells in the preripheral blood as a function of donor age.

In order to test the age-dependent malignancy of transformed hematopoietic progenitor cells *in vivo* in the least biased manner possible, and to exclude any potential non cell-autonomous, age-related, effect on the disease evolution, bone marrow cells were purified from CombitTA-BCR-ABLp190 sacrificed mice in which *BCR-ABL* expression had been kept repressed all life. Then, cells from donors sacrificed at either 4-, 12- or 20-months were injected intravenously into syngenic host mice of 4 months of age. This transplantation into a non doxicyclin-treated recipient lead to transgene derepression and expression of the oncogene in a simultaneous manner. 100% of the injected mice developed B-ALL with all the phenotypic characteristics previously described for the CombitTA-BCR-ABLp190 mice [20], namely the presence in the peripheral blood of organ-infiltrating blast cells co-expressing B-cell and myeloid markers (Figure 3). However, B-ALL derived from 12- and 20-month transformed HSCs could be distinguished by two distinct but interrelated relevant features: first, 20-month-ALLs presented increased cellularity of B220, Mac1 co-expressing blasts (Figure 3B-C). Second, and most importantly, animals with B-ALL derived from 4-month-old donors survived nearly twice as long as those with B-ALL from 12- and 20-month-old donors (88 versus 50.5 and 33 days, respectively; log rank *P* < 0.0001 and 0.0001, respectively; Figure 3A). These findings therefore prove that the malignancy of B-ALL increases with the age of the leukemic-cell-of-origin (i.e., with the age of the normal progenitors from which the disease arises). Leukaemias originating from older progenitors present a more aggressive phenotype, and have a much faster evolution, than leukaemias initiating in young progenitors. Our results also show that, to a great extent, disease evolution is programmed from the beginning, since the starting cell determines the aggressiveness of the phenotype, and also that the decline of the immune response with age is not responsible for the increased development or aggressiveness of the tumours in the elderly. In agreement with these observations it has been recently shown, using a retroviral transduction system, that aged B-cell progenitors present a reduced fitness due to impaired signaling machinery and that this, since the oncogene covers in part the need for kinase signaling, promotes the positive selection for BCR-ABL^+^ cells [21].

In summary, our study of B-ALL provides the first *in vivo* experimental evidence, using a conditional transgenic mouse model, showing that an increase in the age at which transformation occurs has a direct impact in the malignant potential of cancer cells. This is in agreement with clinical observations showing that age-related cell intrinsic factors may play a leading role in the impact of age on B-ALL malignancy [3]. Also, these findings further support the clinical relevance of this model and its uselfulness for future studies to determine how specific oncogenic mechanisms affect age-related malignancy, and to determine how age-related cell-extrinsic host mechanisms such as immune function and others impact B-ALL malignancy.

## METHODS

### Cell culture

Cell lines used include Ba/F3 cells expressing the human protein BCR−ABL^p190^(Ba/F3-p190) [19]. Cells were maintained in Dulbecco's modified Eagle's medium (DMEM) supplemented with 10% fetal calf serum (FCS).

### Mice and transplantation experiments

The CombitTA-*BCR*-*ABL^p190^*mice have been previously described [20]. In order to investigate the impact of age of the target cells in B-ALL malignancy, bone marrow transplantation experiments were performed. BM cells were isolated and highly purified from either 4-, 12- and 20-month old CombitTA-p190 mice that had been kept on doxicycline water for all their life. Marrow cells were flushed from the femurs with a syringe containing 2 mL PBS-1% FBS. In each cohort these BM cells were injected into the tail vein of the irradiated recipient syngenic mice (4 Gy) at 1 × 10^6^ cells per mouse. All recipients were maintained in microisolator cages on sterilized food and acidified sterile water. Diseased recipient mice were sacrificed and assessed for B-cell leukaemia development. All experiments were done according to the relevant regulatory standards.

### Flow cytometry

Nucleated cells were obtained from total mouse bone marrow (flushing from the long bones), peripheral blood, thymus, liver or spleen. In order to prepare cells for flow cytometry, contaminating red blood cells were lysed with RCLB lysis buffer and the remaining cells were then washed in PBS with 2% FCS. After staining, all cells were washed once in PBS with 2% FCS containing 2 mg/mL propidium iodide (PI) to allow dead cells to be excluded from both analyses and sorting procedures. Monoclonal antibodies were obtained from Pharmingen. The samples and the data were analyzed in a FACSCalibur using the CellQuest software (Becton Dickinson). Specific fluorescence of FITC and PE excited at 488 nm (0.4 W) and 633 nm (30 mW), respectively, as well as known forward and orthogonal light scattering properties of mouse cells were used to establish gates. Unspecific antibody binding was suppressed by preincubation of cells with CD16/CD32 Fc-block solution (BD Pharmingen). For each analysis, a total of at least 5.000 viable (PI-) cells were assessed.

### Real-time PCR quantification

To analyze expression of CombitTA-*p190* in Sca1^+^Lin^−^ cells, total RNA was prepared using the Trizol Reagent (Gibco-BRL). Reverse transcription (with random hexamer primers) was performed as described [22]. Real-time quantitative PCR was carried out for the quantitation of CombitTA-*p190*. Fluorogenic PCRs were set up in a reaction volume of 50 ml using the TaqMan PCR Core Reagent kit (PE Biosystems). cDNA amplifications were carried out using the same primers in a 96-well reaction plate format in a PE Applied Biosystems 5700 Sequence Detector. Thermal cycling was initiated with a first denaturation step of 10 min at 95°C. The subsequent thermal profile was 40 cycles of 95°C for 15 s, 56°C for 30 s, 72°C for 1 min. Multiple negative water blanks were tested and a calibration curve determined in parallel with each analysis. The *abl* endogenous control (PE Biosystem) was included to relate CombitTA-*p190*to total cDNA in each sample. The sequences of the specific primers and probe were as follow: *BCR*-*ABL^p190^*, sense primer 5'-CCGCAAGACCGGGCAGAT −3', antisense primer 5'-CAGATGCTACTGGCCGCT GA-3' and probe 5'-TGGCCCAACGATGGCGAGGG-3'; c-Abl, sense primer 5'-CACTCTCAGCATCACTA AAGGTGAA-3', antisense primer 5'-CGTTTGGGCT TCACACCATT-3', and probe 5'-CCGGGTCTTGGGTTATAATCACAATG-3'.

### Statistical analysis

Statistical analysis of differences in Kaplan-Meier survival plots was performed using the Log-rank (Mantel-Cox) test.
